# Data of adsorption of Basic Blue 41 dye from aqueous solutions by activated carbon prepared from filamentous algae

**DOI:** 10.1016/j.dib.2018.10.023

**Published:** 2018-10-09

**Authors:** Shirin Afshin, S.Ahmad Mokhtari, Mehdi Vosoughi, Hadi Sadeghi, Yousef Rashtbari

**Affiliations:** a Department of Environmental Health Engineering, School of Health, Ardabil University of Medical Sciences, Ardabil, Iran; b Social Determinants of Health Research Center, School of Health, Ardabil University of Medical Sciences, Ardabil, Iran; c Students Research Committee, Faculty of Health, Ardabil University of Medical Sciences, Ardabil, Iran

**Keywords:** Adsorption, Basic Blue 41, Activated carbon, Filamentous algae

## Abstract

For this data article the adsorption of Basic Blue 41 (BB 41) dye by activated carbon derived from filamentous algae (AAC) of available in agriculture waste as cheap adsorbents were examined. Activated carbon has been widely used as an adsorbent regard to its massive specific surface area, high porosity, reusability and thermal stability for the removal of pollutants from effluent. These filamentous algae grow widely in irrigation streams, causing decreasing speed of water flow and are not even eaten by livestock so are considered agricultural wastes. They can be used as precursors for activated carbon preparation and as adsorbent for the dye removal. The data of initial dye concentration (50–200 mg//L), pH of dye solution (3–9), adsorbent dosage (0.25–2 g/L), and contact time (5–200 min), were assessed. The structure of AAC was characterized by X-ray diffraction and Fourier transforms infrared spectroscopy. Activated carbon with a 94% removal of dye at concentration of 100 mg/L, pH 9, and adsorbent dose 1 g/L after 90 min. The data of isotherms and Kinetics indicated that the experimental data are fitted to Langmuir and second-pseudo-order models. Under the optimum conditions, maximum adsorption capacity of the AAC in Langmuir model enhanced to amount of 125 mg/g. According to the experimental data, filamentous algae are a suitable raw material for activated carbon production.

**Specifications table**

**Table:** 

Subject area	Environmental Engineering
More specific subject area	Wastewater technology
Type of data	Tables, Figures and Images
How data was acquired	Alga powdered was used to preparation activated carbon.
	All experiments were done using UV–vis spectrophotometer (model DR 5000, HACH). Characterization of structure AAC were determined by Philips X’Pert Pro instrument for X-ray Powder Diffraction (Netherlands) and Fourier transforms infrared (PerkinElmer, USA). The pH meter (Sense Ion 378, Hack model) and centrifuge (Eppendorf versatile 5810 model) were used.
	The obtained data were analyzed using isotherm and kinetic models.
Data format	Analyzed
Experimental factors	All dyes samples were performed in contact time and various solution pH at room temperature.
Experimental features	The optimum contact time and pH were determined for adsorption of BB 41 from aqueous solutions by AAC.
Data source location	Ardabil city, Ardabil province, Iran
Data accessibility	Data are included in this article
Related research article	J. Torres-Pérez, Y. Huang, P. Hadi, H. Mackey, G. McKay, Equilibrium, Kinetic and Optimization Studies for the Adsorption of Tartrazine in Water onto Activated Carbon from Pecan Nut Shells, Water, Air, and Soil Pollution. 229 (2018) 73 [1].
	I.A.W. Tan, A.L. Ahmad, B.H. Hameed, Adsorption of basic dye on high-surface-area activated carbon prepared from coconut husk: Equilibrium, kinetic and thermodynamic studies, J. Hazard. Mater. 154 (2008) 337–346 [2].
	A.B. Leite, C. Saucier, E.C. Lima, G.S. dos Reis, C.S. Umpierres, B.L. Mello, M. Shirmardi, S.L.P. Dias, C.H. Sampaio, Activated carbons from avocado seed: optimisation and application for removal of several emerging organic compounds, Environ. Sci. Pollut. Res. 25 (2018) 7647–7661 [3].

**Value of the data**

• The data provides information about the adsorption process of BB 41 dye from aqueous solution by activated carbon under room temperature.
• Activation method can be used to prepare activated carbon from algae powdered using H_3_PO_4_ and data will be advantageous for scale up and design the adsorption experimental set up for removing dyes.
• The adsorption process by activated carbon can be used as a convenient and environmentally friendly method for removing of different concentrations of dye.

## 1 Data

**Fig. 1:**
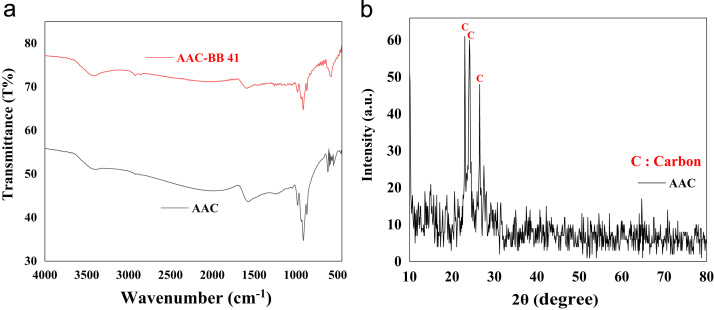
FTIR (a) and XRD (b) patterns of AAC.

**Fig. 2:**
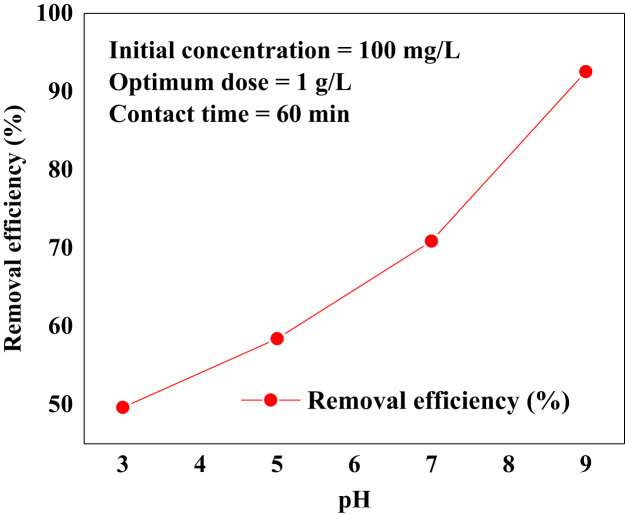
Data of initial pH on the adsorption of BB 41 by the AAC.

**Fig. 3:**
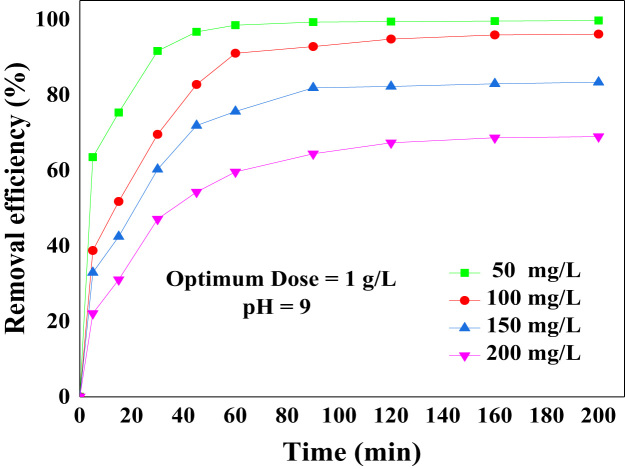
Data of the contact time on the adsorption of BB 41 by AAC.

FTIR spectra was recorded in the range 400–4000 cm^−1^ using a Fourier transform infrared spectrometer (PerkinElmer, USA), and Philips X’Pert Pro instrument (the Netherlands) was used to get XRD patterns of the activated carbon, respectively. The obtained data are shown in Fig. 1(a) and (b). The data of pH solution and contact time on removal efficiency are presented in Figs. 2 and 3. The kinetic and isotherm data and equation are calculated and listed in Table 1, Table 2, Table 3. Comparison the adsorption capacity of AAC with the other adsorbents is presented in Table 4.

**Table 1:** Equations of isotherm and kinetic models [11], [12], [13], [14].

**Model types**	**Model name**	**Equation**
Isotherm models	Langmuir	1/*q*_e_ = 1/(*q*_m_*K*_1_*C*_e_) + (1/*q*_m_)
	Freundlich	Log *q*_e_ = log *k*_f_ + (1/*n*)log *C*_e_
Kinetic models	Pseudo-first-order	Log(*q*_e_−*q*_t_) = log *q*_e_−(*k*_1_/2.303)*t*
	Pseudo-second-order	*t*/*q*_e_ = 1/(*k*_2_$q_{e}^{2}$) + 1(1/*q*_e_)*t*

**Table 2:** Kinetic parameters of BB 41 adsorption onto AAC obtained using of the pseudo-first order and pseudo-second order models [11].

**Model**	**Pseudo-first-order**				**Pseudo-second-order**		
*C*_o_ (mg/L)	*q*_e_,_exp_ (mg/g)	*k*_1_ (1/min)	*q*_e_,_cal_ (mg/g)	*R*^2^	*k*_2_ (g/mg/min)	*q*_e_,_cal_ (mg/g)	*R*^2^
50	50	0.026	12.07	0.8644	0.0083	50.505	0.9996
100	96.4	0.028	59.08	0.979	0.0012	100	0.9968
150	124	0.026	73.89	0.9404	0.0008	131.57	0.9962
200	137.9	0.031	125.37	0.9796	0.0004	147.05	0.994

**Table 3:** Langmuir and Freundlich isotherms parameters for adsorption of BB 41 onto AAC [15], [16], [17].

**Isotherm model**	**Langmuir isotherm**			**Freundlich isotherm**		
**Parameter**	*K*_L_ (L/mg)	*q*_m_ (mg/g)	*R*^2^	*K*_f_ [(mg/g) (mg/L)1/*n*]	*n*	*R*^2^
**Value**	1.818182	125	0.9574	63.88516	6.325111	0.9493

**Table 4:** Comparison of the adsorption capacity of BB 41 onto various adsorbents.

**Adsorbents**	***q***_**m**_**(mg/g)**	**References**
Sodium alginate	12	[18]
Raw rice husk	24.4	[19]
Modified rice husk	34.6	[19]
Untreated antibiotic waste	111	[20]
N, F-codoped flower-like TiO_2_	143	[21]
Activated carbon	125	This data article

## 2 Experimental design, materials and methods

### 2.1 Materials

For this data article, all chemicals materials were purchased from Merck in analytical grade. Basic Blue 41 (Empirical Formula: C_20_H_26_N_4_O_6_S_2_, purity ≥ 98%) was the commercial product supplied from Alvan Sabet Hamadan Co (Iran) and used without further purification. The algae were collected from river in Ardabil, Iran. The pH of the solution was adjusted by mixing with the appropriate amount of 0.1 M (H_2_SO_4_/NaOH). A stock solution of dye solutions with a concentration of 1000 mg/L was prepared by dissolving the required quantity of the dye in distilled water. Distilled water was used throughout in all the experiments.

### 2.2 Preparation of activated carbon

The algae were collected from the streams in Ardabil, Iran. The algae were washed with using distilled water several times to remove the contamination and impurities such as dust and stones. The algae were dried at 60 °C in oven for 72 h. Then dried algae were powdered to size 100 meshes. Chemical activation of the powdered precursor was accomplished with phosphoric acid (H_3_PO_4_). Powdered algae were impregnated with 30 wt% diluted H_3_PO_4_ in 3:1 (activating agent/ powdered algae) ratio and soak time of one hour. The achieved product was placed in an electric furnace (5 °C/min) for 3 h at 650 °C in a nitrogen flow rate of 94.4 mL/min. The samples were washed with HCl, hot water and distilled water respectively, to remove residual organic and mineral matters, and then dried in an oven at 105 °C for 2 h. At the final preparation step, activated carbon samples were crushed and sieved in mesh size 100 to obtain homogenous particle size [4], [5].

### 2.3 Experimental procedure

Batch adsorption experiments were performed in 100 mL Erlenmeyer, in different concentrations of the BB 41 solutions and requested amount of AAC were placed. The pH of the solution was adjusted by mixing with the appropriate amount of 0.1 M H_2_SO_4_ or NaOH. Then a specific amount of adsorbent was added to the Erlenmeyer flasks. The mixtures were continuously shaken in different times (agitation rate = 250 rpm) at room temperature. After adsorption experiments, immediately the suspensions were separated by centrifuge. Then concentration of the remaining BB 41 was analyzed with a UV–vis spectrophotometer by monitoring the intensity of the peaks at wavelength of 617 nm [6] and comparing the values with those in the calibration curve. The experiments were repeated three times in during the process. The adsorption rate *R* (%) and the equilibrium adsorption capacity *q*_e_ (mg/g) were calculated using the Eqs. (1) and (2), respectively [7], [8], [9], [10].

(1) $$Removal\;Percentage\;(\%)=(\frac{C_{0}-C_{e}}{C_{0}})\times 100$$

(2) $$Adsorption\;capacity,\;q_{e}=\frac{(C_{o}-C_{e})\times V}{M}$$

Where *C*_O_ and *C*_e_ are the initial and the final concentration of BB 41 (mg/L), respectively, *V* is the volume of dye solution (L), and *M* is the dry weight of adsorbent used (g).
